# Anaesthetic Management of a Foramen Magnum Meningioma Resection Surgery: A Case Report

**DOI:** 10.7759/cureus.37336

**Published:** 2023-04-09

**Authors:** Daniel Rodrigues, Cidália Marques, João Marques, Maria Antunes, Adriano Moreira

**Affiliations:** 1 Department of Anesthesiology, Centro Hospitalar Universitário de São João, Porto, PRT; 2 Department of Anesthesiology, Hospital da Senhora da Oliveira, Guimarães, PRT

**Keywords:** sitting position, neurosurgery, meningioma, foramen magnum, air embolism

## Abstract

The foramen magnum meningioma (FMM) is one of the most threatening tumours among the meningiomas because of its specific location, clinical course, subtle onset, and relatively big dimensions at presentation.^ ^Tumour size may mandate careful airway management to avoid further brainstem compression. The surgical management of these complex tumours in the posterior fossa can be performed with the patient in several positions. A lot of surgeons believe the sitting position provides important advantages, yet this remains controversial. We report a successful approach to a large FMM resection surgery performed in the sitting position.

## Introduction

Meningiomas represent 13%-26% of all intracranial tumours, and among them, foramen magnum meningiomas (FMMs) compose 2.6% [1]. However, FMMs are important due to their unique location and potential complications. Their indolent expansion at the craniospinal junction makes the diagnosis complex, and they are often only detected when large. As they grow, they invariably lead to brainstem compression; thus, atlanto-occipital extension during airway manoeuvres must be avoided in bigger dimension tumours to prevent further compression. FMM surgery is an important, and complicated, first step in the management of symptomatic tumours, and it can be performed in several positions. Although the sitting position is preferred by a lot of neurosurgeons, it adds some special care and requires monitoring for complications such as venous air embolism or paradoxical air embolism [2]. During the intraoperative period, the anaesthesiology team’s goals are to preserve the brain and general patient physiology and facilitate surgery by correcting any adverse effects of the surgery and underlying pathology. This requires adequate patient monitoring, cardiorespiratory support, management of fluid therapy, and knowledge about the effects of anaesthetic agents on brain physiology.

## Case presentation

**Figure 1:**
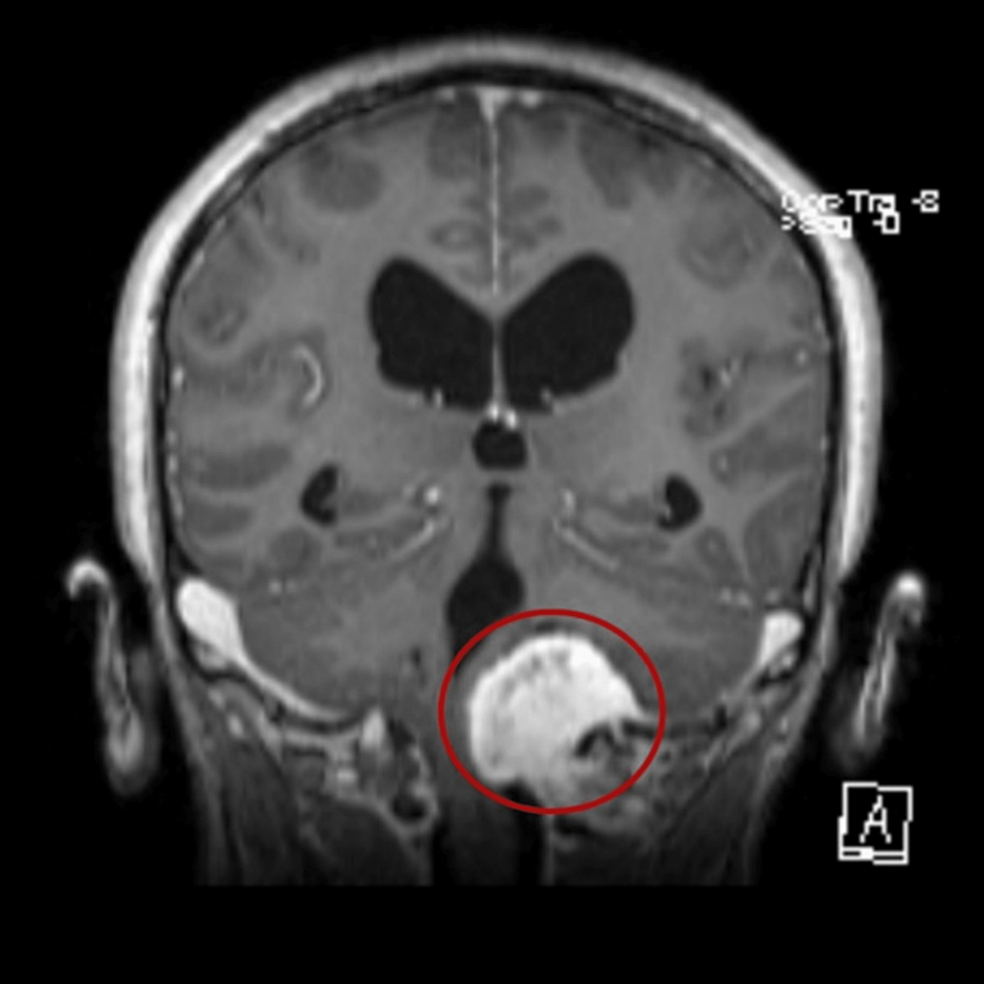
Cranioencephalic MRI revealing a spinoclival tumour with 36 mm in its bigger axis (red circle). MRI: magnetic resonance imaging.

A 52-year-old woman, classified with a physical status of II according to the American Society of Anaesthesiologists (ASA), was admitted for FMM resection under total intravenous anaesthesia (TIVA). She was a smoker and had allergic rhinitis. The patient presented to the emergency department, one month earlier, with an occipital headache associated with dizziness, particularly when standing up or flexing the upper body or head, for the previous three weeks. She also complained of gait difficulties and episodes of vomiting during the same period. The worsening of her symptoms prompted her visit to the emergency department. On neurological examination, only mild left upper limb discrepancy on the finger-to-nose test and gait abnormalities were found. A computed tomography scan was performed, revealing a lesion at the foramen magnum extending upwards. The tumour had its origin below the foramen magnum and grew upwards, making it spinoclival. It measured 36 mm at the anteroposterior and bigger axis (Figure 1). Written informed consent was obtained from the patient for the publication of this case report, including any accompanying images or other materials.

Due to the tumour size, to avoid atlanto-occipital overextension, before induction, the patient was sedated using a Minto model remifentanil target-controlled infusion (TCI), initiated with a target effect-site concentration of 1 ng/mL, adjusted as needed, and with the help of flexible airway endoscopy and adequate airway topicalisation with lidocaine 1%, the patient was intubated. After intubation, a propofol Schneider TCI was initiated with a target effect-site concentration of 3 mcg/mL and the remifentanil infusion was increased to 3 ng/mL. An arterial line in the radial artery and a multipore central venous catheter (CVC) in the subclavian vein were placed. Afterwards, the patient was positioned in the sitting position. Along with the surgery, the propofol and remifentanil infusions were titrated as needed to attain, respectively, a target Bispectral Index™ (BIS™; Medtronic, Minneapolis, MN, United States) of 40-60 and an adequate block of the nociceptive stimulus evaluated by the patient's haemodynamic response. During the application of the pins from the head fixation device, a three- to four-fold pre-emptive increase in the remifentanil target concentration is required to avoid a dramatic sympathetic response with severe hypertension and tachycardia. Throughout the procedure, the patient was monitored with the ASA standard monitoring, central venous pressure, direct arterial pressures, cerebral oximetry with INVOS™ (Medtronic, Minneapolis, MN, United States), a near-infrared spectroscopy-based device, processed electroencephalogram with BIS™, precordial Doppler, and somatosensory and motor evoked potentials (SSEP and MEP).

Before surgical incision, to reduce cerebral oedema and tumour tension, 4 mg of dexamethasone, 25 g of mannitol, and 40 mL of 20% saline were administered. Antibiotic surgical prophylaxis was performed with 400 mg of teicoplanin.

The surgery proceeded without complications, and the patient remained haemodynamically stable throughout the nine-hour procedure. The patient had a total urine output of 1275 mL (approximately 142 mL/h), measured over the course of the procedure. Goal-directed fluid therapy was used to target normovolaemia using haemodynamic parameters such as maintaining the pulse pressure variation below 13%. To achieve this, the patient received 1000 mL of 0.9% saline solution and 1000 mL of Ringer's lactate. Overall, the patient's fluid management was carefully monitored and adjusted to maintain haemodynamic stability.

**Figure 2:**
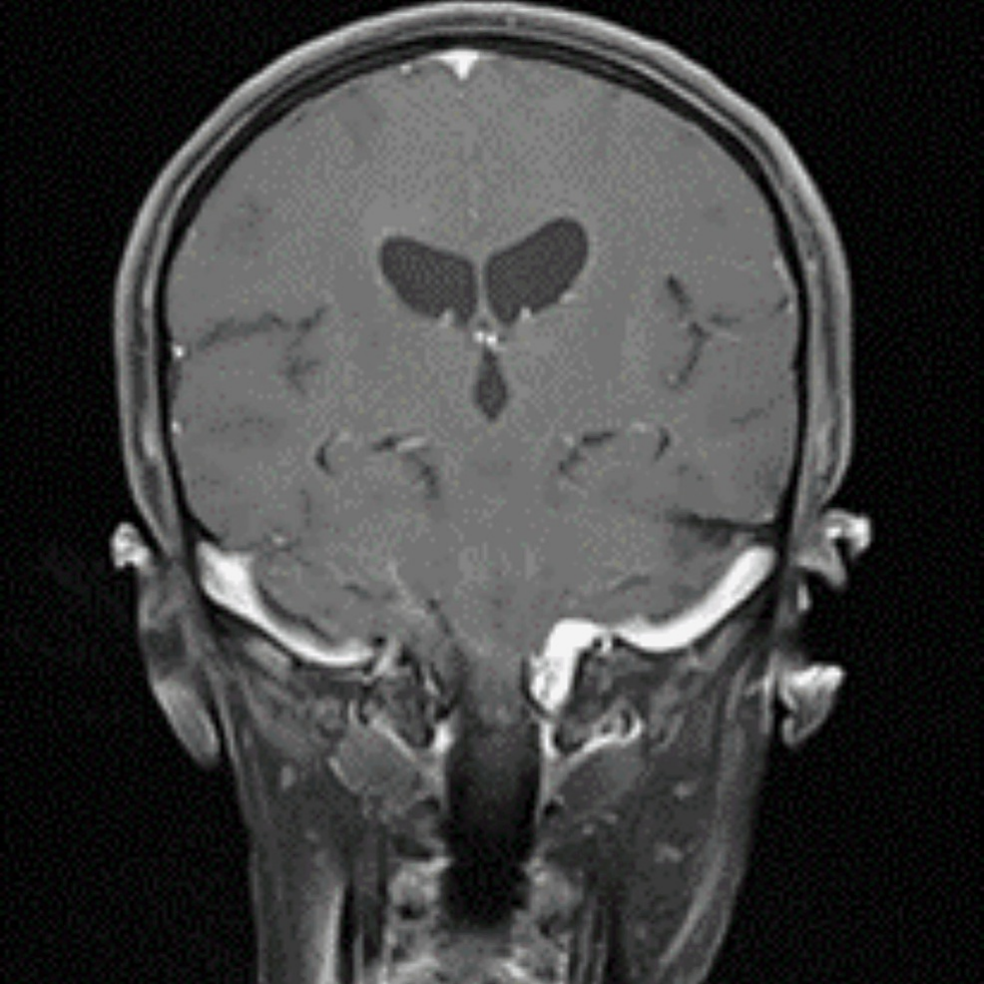
Post-operative cranioencephalic MRI. MRI: magnetic resonance imaging.

Afterwards, the patient was transported, mechanically ventilated and under sedation, to the neurosurgery intensive care unit (ICU). The post-operative magnetic resonance imaging (MRI) showed complete removal of the lesion (Figure 2). She was discharged 21 days after surgery and readmitted three days after discharge due to transverse sinus thrombosis and a cerebrospinal fluid fistula to the skin that were treated during a second 20-day hospitalisation. 

## Discussion

FMMs are rare, making it difficult for anaesthesiologists and neurosurgeons to achieve great levels of experience in their management during surgical resection. Before the era of modern microsurgery and neuroanesthesia, the mortality rate after FMM resection was very high, up to 45% in some series; currently, the mortality rate is around 6.2% [1]. The current advances in microsurgical techniques and anaesthesia procedures, improvements in perioperative care, and increased experience of neurosurgeons and anaesthesiologists with FMM patients have resulted in better outcomes and low mortality rates [1].

FMM resection surgery is a cornerstone procedure in the management of these tumours. It can be performed in several positions, depending on the surgeon’s approach and tumour location. Although many surgeons highlight important advantages of the sitting position, such as offering the best surgical access and anatomic orientation, as well as gravitational venous and cerebrospinal fluid drainage away from the surgical site, it has troublesome aspects, namely the bigger risk of cerebral hypoperfusion, tension pneumocephalus, or air embolism [3].

A reduced end-tidal carbon dioxide (CO_2_) may be one of the first indicators of air emboli in patients under anaesthesia. On the other hand, a decreased oxygen saturation on pulse oximetry is regarded as a late sign of vascular air emboli. Electrocardiogram tracings in the presence of air embolism may show tachycardia, ST segment changes, or evidence of right heart strain [4]. Because these signs are unspecific and may not be present when these complications occur, the use of precordial Doppler for early detection and a multipore CVC for intervention are mandatory. Regarding the monitoring of these events, transesophageal echocardiography is capable of detecting very small volumes of air, but its usefulness is dependent on the anaesthesiologist’s acquaintance with the technique.

Due to the risk of paradoxical air embolisms, which can be fatal, pre-operative screening with an echocardiogram is mandatory to detect any unknown intracardiac shunts in patients proposed for suboccipital surgery in the sitting position. If present, surgical closure should be discussed with cardiology and performed if possible. If not feasible, these patients should not be candidates for the sitting position, and a prone or 3/4 prone positioning should be used.

Regarding air embolisms, intravascular hydration is thought to help increase the patient’s venous pressure and reduce the risk of air entrainment [3]. If suspected, air emboli should be promptly treated with 100% oxygen, increased fresh gas flows, and interruption of nitrous oxide if in use. The surgeon should be informed so that any possible source of air entrance can be detected, the field can be sealed with saline, and sealant can be placed over any open sinuses. If it is safe to do so, the patient's head should be lowered as much as possible, preferably below the level of the heart, to increase cerebral venous pressure. Aspiration from a multipore CVC inside or close to the right atrium can assist with air extraction [3].

Other considerations of the sitting position are the important complications that may occur from excessive flexion of the head, namely laryngeal dysfunction, dysphagia, and swelling and necrosis of the tongue. To avoid these, a minimum distance of 3 cm between the patient’s mentum and thorax is recommended.

As the tumour grows, it unfailingly leads to brainstem compression. From an anaesthetic point of view, the degree of brainstem compression has different implications. During airway management, atlanto-occipital extension may worsen brainstem compression, and consequently, in patients with big tumours, the airway should be secured with minimal cervical mobilisation, namely with flexible airway endoscopy or videolaryngoscopy. Depending on the tumour size, origin, and severity of brainstem compression, osmotic agents and corticosteroids are administered to reduce cerebral oedema and the tumour’s tension. The most frequently used osmotic agents are mannitol and 20% saline, which were the ones used in this case.

Monitoring with SSEP and MEP allows detection of early lesions or suffering of the brainstem and is intended to aid the neurosurgeon in the preservation of neurological function. SSEP provide a measure of ascending pathways; on the other hand, the function of the ninth and 10th cranial nerves can be monitored with endotracheal tubes that monitor the laryngeal muscles and vocal cord contraction, while electromyographic recordings of the sternocleidomastoid muscle and tongue, respectively, reflect the activity of 11th and 12th cranial nerves. If any one of these techniques exhibits a change, the surgeon must be warned of a potentially dangerous manoeuvre and should pursue a distinct approach regarding dissection [5]. Bradycardia may occur and should be treated with atropine.

Despite these advantages, the monitoring of SSEP and MEP precludes the use of volatile halogenated anaesthetics and the use of neuromuscular blockers, leaving the anaesthesiologist with few options besides the TIVA without curarisation. Neuromuscular blockers may be used for intubation. In that case, if depolarising agents are used, their effect will wear off after a single bolus; if non-depolarising agents are used, their effect must be reversed in order to avoid interference with the evoked potentials. It is also important to be aware that the stimulation of the 12th cranial nerve will occasionally cause protrusion of the tongue, which, if not returned to position by the anaesthesia staff, may cause post-operative swelling [5]. Therefore, if the 12th cranial nerve is being monitored, a double bite block should be used and secured. 

As anticipated, strong and effective communication between the neurosurgery, anaesthesiology, nursing, and neurological monitoring teams is imperative to guarantee that all the complications are anticipated, averted, and rapidly handled if they occur.

## Conclusions

FMM resections are complex surgeries with specific complications, which require special care, especially when performed in the sitting position. In order to guarantee the best outcomes for the patients, these complications need to be avoided and promptly treated.

Although rare, the sharing of information through case reports and series of cases, along with the development of surgery techniques and anaesthesia procedures, has allowed a drastic reduction in the mortality rate. Further improvements in the approach to these tumours are required, making additional studies pertinent.
